# Improvement of the quality payment program by improving data reporting process: an action research

**DOI:** 10.1186/s12913-018-3472-4

**Published:** 2018-09-06

**Authors:** Shabnam Iezadi, Jafar Sadegh Tabrizi, Akbar Ghiasi, Mostafa Farahbakhsh, Kamal Gholipour

**Affiliations:** 1 Social Determinants of Health Research Center, Health Management and Safety Promotion Research Institute, Tabriz University of Medical Sciences, Tabriz, Iran; 2 Tabriz Health Service Management Research Center, Health Management and Safety Promotion Research Institute, Tabriz University of Medical Sciences, Tabriz, Iran; 3 Department of Health Services Administration, School of Health Professions, The University of Alabama at Birmingham, Birmingham, USA; 4 Road Traffic Injury Research Center, Tabriz University of Medical Sciences, Tabriz, Iran; 5 Iranian Center of Excellence in Health Management, School of Management and Medical Informatics, Tabriz University of Medical Sciences, Tabriz, Iran

**Keywords:** Pay-for-quality, Pay-for-performance, Action research, Primary health center, Data reporting

## Abstract

**Background:**

Successful implementation of pay-for-quality (P4Q) programs mostly depends upon a valid, timely, and reliable data about quality measures generated by providers, and interpreted by payers. The aim of this study was to establish a data reporting method for P4Q program through an action research.

**Methods:**

Qualitative method was used to align theory with action through a three-cycle action research. The study was conducted in September 15, 2015 to March 15, 2017, in East-Azerbaijan, Iran. The purposeful sampling was used to select participants. The participants included healthcare providers, staff in district health centers (DHC), experts, and managers in the provincial primary health center (PPHC). Data was collected by interviews, focus group discussions, and expert panels. Content analysis was used to synthesize the data. In each step, decisions about data reporting methods were made through a consensus of expert panel members.

**Results:**

The most important dimensions of data reporting method were data entry and accuracy, data reporting, data analysis and interpretations, the flexibility of method, and training. By establishment of an online data reporting system for the P4Q program, a major improvement was observed in the documentation of performance data, the satisfaction of health care providers and staff (e.g. either in DHCs or PPHC), improvement of the P4Q program and acceptance of the P4Q program by providers. Following the present study, the online system was expanded in Iran’s public health system for data collection and estimating the amount of incentive payments in P4Q program. Moreover, more improvements were achieved by linking the system to EMRs and also, providing automated feedback to providers about their own performance.

**Conclusions:**

A web-based computerized system with the capability of linking medical record and also its ability to provide feedback to healthcare providers was identified as an appropriate method of data reporting in the P4Q program from the viewpoints of participants in this study.

**Electronic supplementary material:**

The online version of this article (10.1186/s12913-018-3472-4) contains supplementary material, which is available to authorized users.

## Background

Recent studies have confirmed the worldwide interest in Pay-for-Quality (P4Q) program as a promising initiative to improve the quality of health care [1–3]. Such interest was launched since the Institute of Medicine (IOM) recommended health plans to use quality-based incentive programs for improving the quality of healthcare, based on established criteria [4, 5]. The P4Q is a quality improvement strategy which uses monetary incentives to improve the quality of healthcare [6]. The P4Q programs have three main elements; measures and performance domains (e.g. incentive payment is based on these measures) payment strategy (e.g. paying incentives for achieving of specific target of measures or improvement of the measures) and data reporting (e.g. method of building the measures and calculating the payment amounts) [7]. Despite widespread usage of the P4Q programs in various health plans, there is no consensus about the best way to design and implement these programs [1, 8].

Data reporting is one of the most important components of a P4Q program, which refers to the process of gathering and interpretation of measures and the evaluation of payments. Successful implementation of the P4Q program mostly depends upon a valid, timely, and reliable data about quality measures generated by providers, and interpreted by payers. The most important influence of a P4Q program is to motivate providers and payers to shift from traditional clinical information systems (paper evaluation) to more automated and computerized systems which can generate data for secondary uses [9, 10].

P4Q programs have improved health information system in different countries; for example, the first implementation of the P4Q program began by “pay-for-reporting” programs in the US. According to that program, physicians were rewarded for just reporting the provided cares. This initiative was a groundwork for the P4Q program [11, 12]. In the UK, acquiring minimal information technology (IT) standards has been designated as an eligibility criterion for participation in the quality payment program [13]. Developing countries such as Iran have infrastructure problems and the use of IT is very limited in healthcare. Some of the main problems about the adoption of IT in Iran’s health system [14] are lack of standardized e-health applications, deployment and training barriers, and privacy and security concerns [15]. Such basic problems affect the successful implementation of programs like the P4Q program that are largely depend on IT for data reporting. Hence, there is an essential need for creating an appropriate data reporting system based on contextual requirements to successfully implement of the P4Q programs. This study aimed to establish data reporting method for the P4Q program in Iran’s public health system based on viewpoints of stakeholders in district health centers (DHCs and PPHC).

### Context

This study was conducted from September 15, 2015, to March 15, 2017, at university-affiliated DHCs in Tabriz which implemented the P4Q program for the first time in Iran. As mentioned in the previous report [16], this program was implemented to improve the incentive payment system for healthcare providers who were not eligible to earn extra money in family physician plan of rural areas. To justify the salary of the health care providers (e.g. who were not eligible to earn extra payments in family physician program), an amount of money was decided to pay as an incentive in the quarterly setting; therefore, the P4Q program was initiated to manage the incentive payment. Data for the P4Q program collected from administrative documents, medical records, and patient surveys. However, making any decision about how to gather and exchange data among healthcare providers and payers was challenging [17]. On the other hand, it was important to involve healthcare providers in the establishment of an appropriate data reporting method due to their direct access to data sources [2]. Studies have shown the importance of the healthcare providers’ participation in designing the P4Q program [18, 19]. For example, Kirschner showed that, participatory action research is an appropriate approach for designing the P4Q program [20]. Due to these reasons and also, in order to establish the best method of data reporting in the P4Q program, a three-cycle action research was implemented with the participation of all stakeholders in planning and implementation of appropriate actions.

## Methods

### Design

**Fig. 1:**
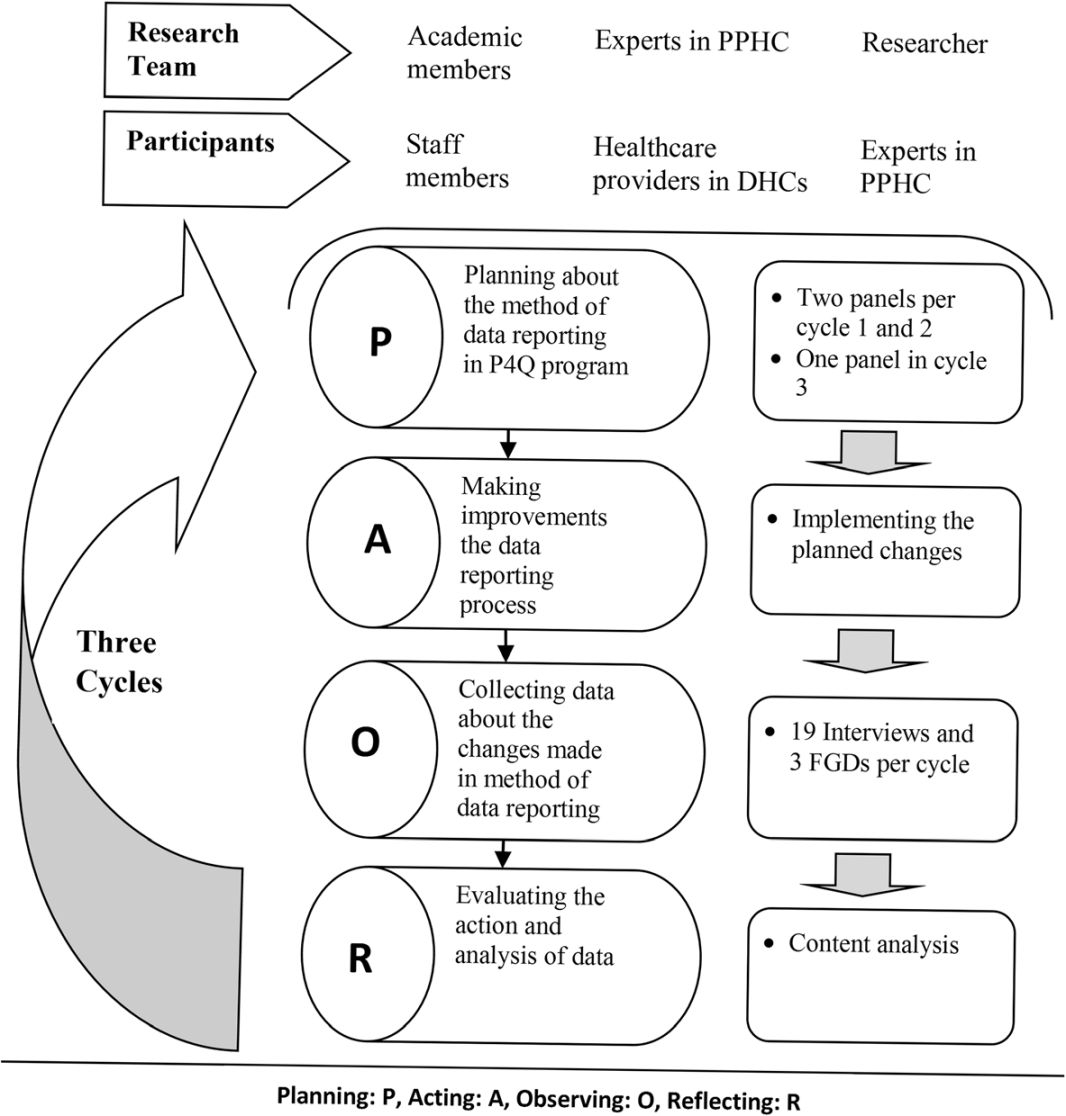
Data collection in four-step participatory action research

A qualitative study with action research approach was used for planning and improving data reporting method in P4Q program in primary health care (PHC) system of East-Azerbaijan, Iran. The research team consisted of two faculty members (Ph.D. in health services management), an expert in PPHC (e.g.MD degree), and two researchers in health system (e.g. Ph.D. and Ph.D. candidate in health services management) (Fig. 1)1. All members of the research team engaged in study design, implementation and reporting the findings. The researchers used Meyer’s Four-step action research framework (e.g.g planning, acting, observing and reflecting) [21] to direct the study. In action research steps, existing documents in DHCs were reviewed in addition to three qualitative methods including interview, focus group discussion (FGD), and expert panel. Although participation in the study was voluntarily, the participants did not allow to tape their interviews. However, two secretaries transcribed independently the whole interviews as well as the main points. At the end of any interview (e.g.either in FGDs or individual interviews) the records of the two secretaries were compared and feedback was provided to participants to correct any potential misconception or misunderstanding.

### Participants

Participants were selected using purposeful sampling. The participants included providers (e.g. from DHCs) and payers (e.g. from PPHC). For interview, the staff of DHCs were included. The inclusion criteria included; the staff authorized for gathering and reporting data to payers in the P4Q program, having more than five years work experience in PHC system, and willing to participate in the study. Managers in PPHC with more than five years of administrative experiences in the PHC system were eligible to participate in the panels. The FGDs participants were selected using purposeful sampling. Two groups of people were eligible to participate in the FGDs; experts in departments of health plan in the PPHC and representatives of health care providers in the DHCs. First, experts in departments of health plans in the PPHC with more than five years of experience in the PHC system became eligible to participate in the FGDs. Due to this criterion, experts in disease management, pharmacy, mental health, occupation and environmental health, and family medicine could participate in the FGD. Second, representatives of health care providers in DHC with more than two years of experience in the PHC system who were willing to participate in the FGDs were included. At the end, participants included 20 healthcare providers, 17 experts in the PPHC, 19 DHC staff, 3 top managers of the PPHC and 4 academic members (Table 1).

**Table 1:** Characteristics of participants in qualitative methods

Data Collection	Number of Participants	Discipline	Age	Work Experience	Education
Interview	19	• Epidemiology (M^a^=2) • Health education (F^b^=2, M = 2) • Family health (F = 4, M = 1) • Public health (M = 4) • Environment health (M = 3) • Occupation health (M = 1)	• 20–30 (*n* = 8) • 31–41 (*n* = 5) • 42–52 (*n* = 4) • 53–63 (*n* = 2)	• 5–10 (*n* = 7) • 11–16 (*n* = 5) • 17–22 (*n* = 4) • 23–28 (*n* = 3)	• Bachelor’s degree (*n* = 13) • Master’s degree (*n* = 6)
FGD					
Healthcare providers in DHCs	20	• Physician (F = 3, M = 1) • Healthcare provider (F = 6) • Teeth technician (F = 3) • Dietitian (F = 2) • Vaccinator (F = 2) • Occupation/Environment health experts (F = 2, M = 1)	• 20–30 (*n* = 10) • 31–41 (*n* = 8) • 42–52 (*n* = 2)	• 5–10 (*n* = 9) • 11–16 (*n* = 7) • 17–22 (*n* = 4)	• Diploma (*n* = 2) • Bachelor’s degree (*n* = 8) • Master’s degree (*n* = 6) • Doctorate (*n* = 4)
Experts in PPHC	17	• Nutrition expert (F = 1) • Family health expert (F = 2) • Communicable disease expert (F = 2) • Non-Communicable Disease expert (F = 1, M = 1) • Health expert (F = 5) • Dentist (M = 1) • Pharmacist (M = 1) • Occupation and environment health experts (F = 2, M = 1)	• 42–52 (*n* = 11) • 53–63 (*n* = 6)	• 17–22 (*n* = 12) • 23–28 (*n* = 5)	• Doctorate (*n* = 9) • Master’s degree (*n* = 8)
EP	7	• Top managers of PPHC (M = 3) • Academic members (F = 1, M = 3)	• 42–52 (*n* = 4) • 53–63 (*n* = 3)	• 17–22 (*n* = 4) • 23–28 (*n* = 3)	• Doctorate (*n* = 7)

^a^*M* Male

^b^*F* Female

### Data collection

Data collection were carried out in the PPHC. The details of each data collection method is explaining in this section.

#### Interview

Total of 57 unstructured and open-ended interviews (19 per cycle) were conducted. The main goal of interviews was to explore the opinions of the DHC’s staff about the most appropriate method of data reporting in the P4Q program (Table 2). Each interview was 30 min on average. The study objectives and corresponding questions were sent to interviewees seven days before each interview to familiarize them with the study objectives (Additional file 1). The date of interviews was double checked with participants. Interviews were implemented by MF (MD) who was the member of the research team and had approximately 20 years’ experience in the PPHC. Two other members of the research team (e.g. SI and KQ, both with Ph.D. degree in health services management) transcribed the interviews.

**Table 2:** Number of qualitative methods used in each cycle of action research

Qualitative Methods	Cycle1	Cycle2	Cycle3	Total
Interview	19	19	19	57
Role	Staff Members in DHCs^a^			
Expert Panel	2	2	1	5
Role	Mangers in PPHC^a^			
FGD	2	2	2	6
Healthcare Providers in DHCs	1	1	1	3
Role	Representatives of Healthcare Providers^a^			
Experts in PPHC	1	1	1	3
Role	Experts in Departments of Health Plans in PPHC^a^			

^a^Participants were the same over the three cycles

#### FGD

The opinions of healthcare providers in the DHCs and experts in the PPHC regarding the best data reporting method in the P4Q program was obtained by conducting nine FGDs. Predetermined and piloted the FGD instruction was used to guide the sessions and conduction the FGD. The questions of the FGD were the same as in interviews. On average, each FGD session was about 45 min. The decision regarding time and place of the FGD sessions were made by agreement between researchers and participants. Face-to-face invitation of the PPHC experts was implemented by a member of the research team and invitation letters were sent subsequently.

In order to increase the probability of participation, at first step, researchers determined the eligible health care providers who might be interested in the FGDs by calling the secretaries of each DHC and explaining the aim of the FGD, eligibility criteria, and introducing the researchers. After finding the eligible participants, the time and place of the FGDs were set up by telephone in the second step. The FGD sessions were guided by the facilitator (MF). Two authors (SI and KQ) assisted the facilitator to direct the sessions. The secretaries also transcribed the interviews independently in each session. Background of the facilitator and secretaries were discussed before. To assure trustworthily, at the end of each session, main points were shared with participants to check for accuracy and quality of the qualitative data.

#### Expert panel

After receiving participants’ ideas in interviews and group discussions about how to eliminate the weaknesses of existing data reporting system, the final decision was made in an expert panel at the end of each cycle.

Totally, five expert panels (e.g. two panels in each of the first and second cycle and one panel in the third cycle) were performed for designing the most appropriate method of data reporting. On average, each panel session was 95 min. At the first cycle of action research, two sessions were conducted to figure out the best method of data reporting in the P4Q program. The panels were guided by the facilitator (e.g. JT, MD, Ph.D. in health services management). In the first session, the facilitator presented an introduction to the project. Then, the panel members discussed the cost of reporting and they determined the least costly data reporting method. However, making a final decision about the most appropriate method of the data reporting was made in the second panel. In the second cycle of action research, possible changes in data reporting method was discussed in a panel session considering the participants’ opinions in the interviews and the FGDs. Three panel members were authorized for reviewing the pros and cons of each change till next session. In the next session, the authorized panel members reported their findings and appropriate changes were applied from the authorized panel members’ recommendations as well as reaching consensus across all panel members. The final panel was held in cycle 3 to address any potential issues of the agreed data reporting method.

### Data analysis

Content analysis method was used to synthesize the qualitative data. The contents were extracted from transcribed interviews. Pairs of the five research team members carefully reviewed the notes several times and coded the notes independently. After that, they compared their cods with each other for any potential contradictions. In case of finding any contradictions, they discussed with each other to mitigate them. In the next step, the pairs of researchers converted the codes into themes and sub-themes. To ensure trustworthiness member checking method was used. Member checking refers to using the individuals that are closest to the situation to validate the findings and interpretations of the researchers [22]. Transcripts created at the end of each interview and the FGDs were shared with interviewees to ensure the accuracy of their opinions. Moreover, the researchers were completely familiar with their tasks and activities during all steps of this research in designing the study, collecting qualitative data, and writing the paper.

## Results

**Fig. 2:**
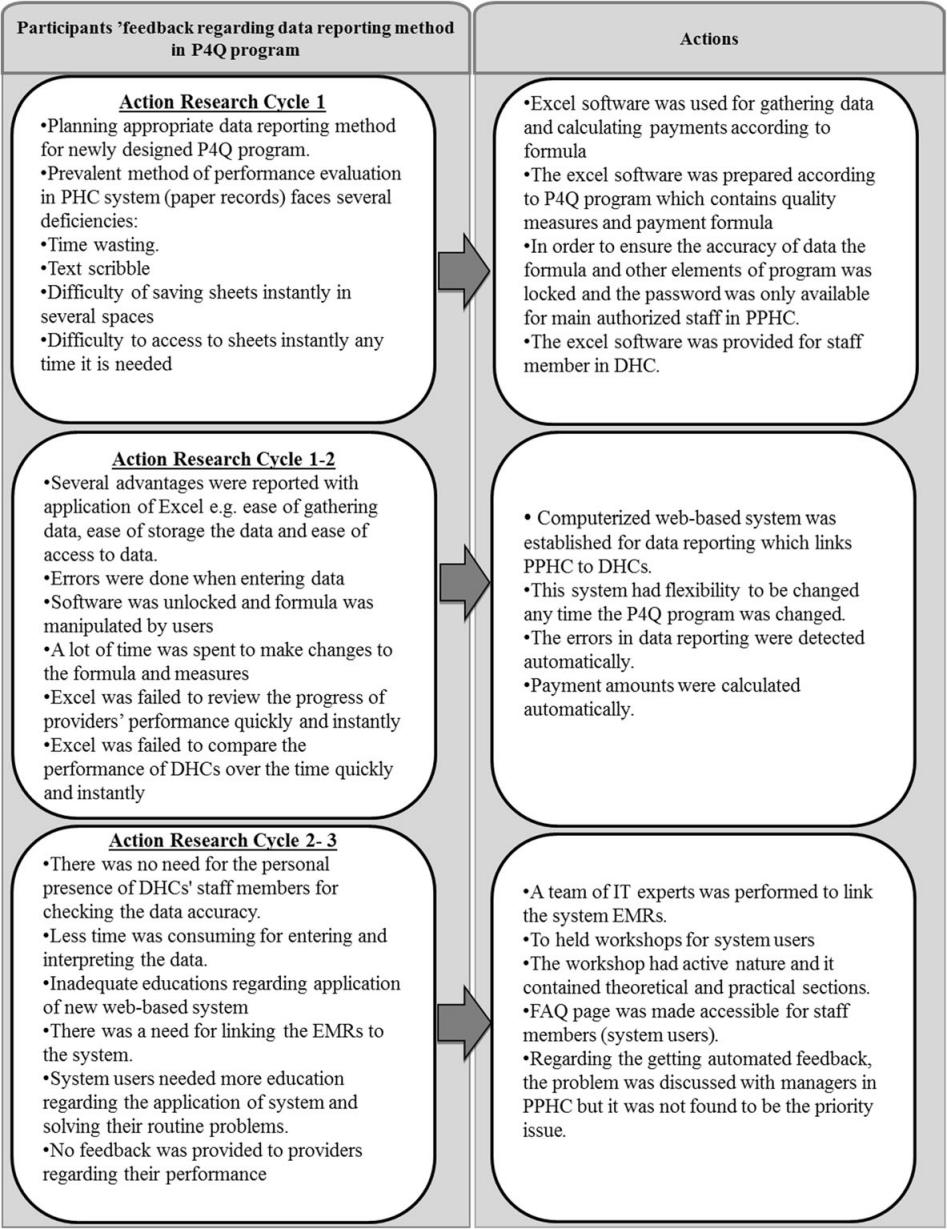
Main findings and actions

The method of data reporting in the P4Q program was identified and established through a participatory process. The research findings and actions are summarized in Fig. 2.

Analysis of initial codes was reported to show how established method of the data reporting in the P4Q program by stakeholders’ participation can improve the acceptance of P4Q program. Participants identified nine themes as the main issues of previous data reporting method in the P4Q program. The findings of interviews and the FGDs were used to eliminate the issues on previous data reporting system for quality payment program. The participants’ statements describing issues as well as potential solutions were reported in the result section. In order to clarification, initials were used; for example, “ARC” for Action Research Cycle (e.g. ARC1, ARC2 or ARC3), participant (e.g. HP = healthcare provider, SM = staff member, E = experts in PPHC, TM = top managers of PPHC and AM = academic member), and data source (e.g. I = interview, FGD = focus group discussion, and EP = expert panel).

### Theme 1: Time-consuming

Excel software was identified as the cheapest and most appropriate tool in an expert panel at the first step of the designing of the data reporting method in the P4Q program. This software is an innovative method of data reporting in East-Azerbaijan and it has several advantages compared to paper sheet such as low cost, ease of application and ease of storage. However, some issues were found in its applicability while using. For instance, a participant pointed out that:

*.. It is true that excel is more comfortable than paper for data reporting. For example, we can save the data files in several storages and the access to the files is easier. But, we are facing some other types of issues. For example, many times, the error occurs in the system and everything gets wrong. Then we have to go PPHC to find out what is the problem. (ARC1 I SM)*

The process was time-consuming for either user staff in DHC or authorized experts in PPHC.

*I have to check the data over and over to find out if there is any mistake. (ARC1 FGD E)*

Using the online web-based system for data reporting saved time for data reporting and data controlling:

*There were some problems while using excel for data reporting. For example, the people who charged for data entering, had reported hanging of the computers and in some cases, deletion of data before saving. Or, sometimes, the power outage was happening and everything was messed up in excel. However, these types of problems disappeared after introducing the online system. (ARC2 I SM).*

*Compared to the previous data reporting method (e.g. paper), the need for error checking by staff in PPHC was declined and data was reported faster than before. (ARC2 FGD E)*

### Theme 2: Manipulation of data

While using Excel as a data reporting method, we designed a table for data collection as well as calculating the amount of payment. We entered the formula in excel and we defined a password to enter. However, the password was unlocked in district health centers, and in some cases, they had changed the original formula. As a result, the amount of payment was calculated more or less than what it was supposed to be.

With the advancement of technology, the ways for manipulation of data even in rigorous software has been increased. Although, the payment formula and targets of measures have been locked in Excel, but sometimes, the formula had been manipulated with unknown reason. Hence, experts in the PPHC had to check all the data and correct the formula when it was necessary:

*I don’t know how they could change the formula. Well, I know it was unintentionally. Anyway, the formula was being manipulated; and so, I couldn’t be sure about data accuracy until checking the whole data again. (ARC2 FGD E)*

Using web-based software resolved this problem:

*Through the new method of the data reporting, any manipulation neither in formula nor in targets of measures were observed. (ARC2 FGD E)*

### Theme 3: Trend analysis in individual/system level

It was impossible to track the progress of healthcare providers’ performance quickly, because, the data was gathered and reported quarterly, and there was separate excel file for each data reporting process.

*The experts should merge several excel files to evaluate the progress of healthcare providers’ performance. However, it was not done in most of the cases! (ARC2 FGD E)*

*In some cases, the score of our centers was low but our progress in a certain period of time was significant. Maybe, our performance was better than other centers in other districts; but, how could they compare our performance?! (ARC2 FGD HP)*

However, using the web-based computerized system for the data reporting has the advantages of tracking the performance of providers, health centers, and also comparing them with others when it is necessary.

*.. I tried to work better because I knew I might be compared to others. (ARC3 FGD HP)*

*A sense of competing was created in DHCs. I thought it might lead to desirable results such as improving the quality of services as one of the main goals of the P4Q program. (ARC3 FGD EP)*

### Theme 4: Feasibility of preparing managerial reports

In traditional methods of the data reporting method like paper and excel, providing the managerial reports regarding the percentage of payment, variation in payment, and providers’ information were problematic.

*We couldn’t routinely provide accurate and timely reports about the percentages of payment. Also, our data was inconsistent about providers’ information in payment due or the time of performance evaluation. Hence, we had to check the data with documented data in the PPHC. That was extra work and time wasting. (ARC1 FGD E)*

In the new web-based system, any change or contrast in provider information alerted and corrected automatically.

*By establishment of the new computerized software, I could provide managerial reports accurately and timely whenever I needed. I only needed to log in to access any data*. *(ARC2 FGD E)*

### Theme 5: Flexibility of data reporting tool

Excel was defined as the appropriate tool for data reporting, because previously, the evaluation process was used to conduct manually (e.g. using paper). The expert needed a more flexible tool like the P4Q. This program is a dynamic process and it is monitored regularly and necessary changes are made in measures, targets, and formula:

*The program changed multiple times and we had to change excel sheets each time. But, we needed a comprehensive tool with a capability of automatic changes due to program changes. (ARC1 EP TM)*

### Theme 6: Training the system users

The ability of training the users was another important aspect of the new system. The staff needed more educations regarding the easiest way of using the system, and solving their problem fast:

*I have not completely learnt the new system. There were some questions remaining unanswered. When I faced any problem, I had to call PPHC and in most of the time, the line was busy and I couldn't contact them when I needed help. (ARC2 I SM)*

In order to solve these issues, three workshops were planned for educating the users in district health centers. Each workshop consisted of a lecture and practical exercises. In practical exercises, participants were divided to six groups and they worked in a computerized system. The interactive nature of the exercises helped the participants to learn from each other. Moreover, a frequently asked questions (FAQ) web page was made available for staff to guide them and to avoid unnecessary contact with the PPHC.

*The workshops were useful for me. Teaching the main issues through lecture and implementing learnings in a real environment helped me to find answers to my questions. (ARC3 I SM)*

### Theme 7: Linking the system to EMRs

As mentioned before, the new web-based system of data reporting solved several issues of Excel software. However, the new system currently does not have the possibility of extracting the data from EMRs. Some participants highlighted this point by saying that:

*My coworkers and I enter the patients’ clinical data into the EMRS and we have to again send the data to our superior for reporting to authorities in DHC after three months. Well, the system is an online then why it does not have the capability to automatically extract data from EMRs! (ARC3 FGD HP).*

Enhancing the capability of the system not only may ease the process of data reporting, but also it would add another advantage for the P4Q program:

*.. If I know that my performance may directly be evaluated by superiors I would work better! So, I would try to enter data more carefully. (ARC3 FGD HP)*

### Theme 8: Providing feedback

In the traditional method, there was no defined mechanisms to link different levels of health system such as provincial, district and health center for monitoring and supervising process. On the other hand, this linkage was established in the web-based system. However, neither the new method nor traditional one had the ability to provide automated feedback to providers. One reason is the privacy policy issue. In fact, according to the PPHC’s policies, several privacy challenges would have emerged if automated feedback had been provided to each healthcare provider. At the first step, it is necessary to promote the capacity of providers and managers.

*Our organizational context is not ready to provide the automated feedback to providers. At the first step, we need to promote the organizational culture. I hope it will be done in the future. (ARC3 EP TM)*

### Theme 9: Acceptance of the program

Increasing the acceptance of the P4Q program was one of the most important achievements of the establishment of data reporting system which designed by contribution of users and healthcare providers. Healthcare providers have more confidence using the web-based method of data reporting compared to the manual methods like paper and Excel. Therefore, higher confidence led to greater acceptance of the quality payment system among participants.

*I trust the web-based system more than manual systems. All the data stored in a major database and payments are calculated automatically. (ARC3 FGD HP)*

*Using the web-based system for data interpretation has made the program more acceptable. (ARC3 FGD E)*

## Discussion

Establishment of the method for data reporting in the P4Q program through participatory action research resulted in more confidence among healthcare providers about the P4Q program in Iran. This study showed that, replacing the data reporting method in the P4Q program by computerized web-based system, improved accuracy of data and acceptance of program through clarifying the program for staff in DHCs. On the other hand, the use of participatory action research provided strong evidence for the implimentation of the new system nationwide. Several studies have suggested the potential benefits of using IT in the P4Q programs. For instance, a trial study by Bardach and colleges in 2017 showed that the P4Q program improved processes and outcomes of cardiovascular care in small practices and those improvements enhanced by adopting electronic health record [23]. Moreover, Robinson et al. (2009) acknowledged that the greater the usage of IT by large practices in the P4Q program and quality improvement programs the better the organizing their practices, balancing experiences in making clinical decisions and interacting with patients [24]. Weiner et al. found that the application of IT in healthcare systems, specifically for measuring and evaluating the performance is greatly a promising tool for achieving desirable clinical outcomes [25]. An action research by Davidson and Heslinga (2007) showed that, IT has a great potential capability to improve the quality of health care, access to health care, and efficiency [26].

From the participants’ standpoint, linking the data reporting system and the EMRs can lead to better documentation of clinical data and ultimately provide more accurate, and timely entered data into the EMRs by healthcare. Fuhlbrigge and colleges (2008) pointed out that reliable payment is available when reliable data are used in the P4Q program [27]. In this respect, another study showed that, the EMR can commonly create more accurate and detailed data in clinical procedures and outcomes than other forms of data like billing data which is provided for payment purpose [2]. Kruse found the improvement in documentation of smoking status through system-wide EMR reminder [28]. Furthermore, a systematic review in 2006 illustrated three main benefits of IT utilization on quality of healthcare including improved adherence to guidelines, better monitoring, and reduction in medication errors, particularly in preventive health areas [29].

The value of this study is related to the context in which the study was implemented. In many developed countries such as the UK and the US, the advanced IT is used for data gathering and reporting. In the UK, the specific computerized system is used for data analysis in the P4Q program [30]. Likewise, in the US, data is extracted from the Healthcare Effectiveness Data and Information Set (HEDIS) and widely used by most of the health plans to measure performance on predefined dimensions of healthcare [31, 32]. However, in developing countries, using such technology is not common. For example, in Egypt, paper documents that is submitted quarterly were used for evaluating the performance in the P4Q program [33]. In Rwanda, documents of monthly activity reports are submitted by facilities to the district steering committee for quarterly payments [34, 35]. Lessons learning from some developing countries can be used in other developing countries to design, implement, and evaluate their own P4Q program. Although developed countries usually do not have these types of problems, developing countries on the other hand, have these types of problems and the result of this study could be helpful to address similar problems in these countries. Another value of this study is related to the implication of the results in national level. Action research provided strong evidence to establishment of data reporting method in the P4Q program for Ministry of Health (MoH). In other words, the insight provided by this study persuaded the MoH to develop the web-based system at the national level.

### Limitations

This study has some limitations. Lack of the quantitative measures to assess the success of actions is the first limitation. This study is a qualitative action research and the authors did not conduct any quantitative analysis. The second limitation is the lack of evaluating the effect of the changes in data reporting method on quality measures. However, the authors reviewed the variations in targeted quality measures in the P4Q program in the period of the study (every 6 months) and overall improvement in most of the measures was seen. But considering the aim of this study, there was no plan to report those results.

## Conclusion

Using a three-cycle participatory action research in designing the method of data reporting in the P4Q program in Iran’s public health system showed major improvement in documentation of data, the satisfaction of health care providers and staff of both the DHC and the PPHC, and improvement of the P4Q program. However, some issues remained unsolved in the new method such as providing automated feedback to healthcare providers regarding their performance and linking the system to the EMRs. The results of this study were applied by experts in the MoH in Iran and the web-based system was expanded in the whole country for gathering the performance data and calculating the payments in the P4Q program. According to the final planned change in cycle3, the IT experts in the MoH are trying to link the web-based system to the EMRs. Additional studies are needed to examine the effects of the new data reporting method on quality and costs.

## Additional files

Additional file 1: Interview protocol. (DOC 26 kb)

Additional file 2: Informed consent letter. (DOC 42 kb)

## Abbreviations

DHC: District Health Center
EMRs: Electronic Medical Records
FAQ: Frequently Asked Questions
FGD: Focus Group Discussion
HEDIS: Healthcare Effectiveness Data and Information Set
IoM: Institute of Medicine
IT: Information Technology
MoH: Ministry of Health
P4Q: Pay-For-Quality
PPHC: Provincial Primary Health Center
