# Supplementary material for: Improvement of the quality payment program by improving data reporting process: an action research
Source: BMC Health Serv Res. 2018 Sep 6;18:692. doi: 10.1186/s12913-018-3472-4 (PMC6128004; doi:10.1186/s12913-018-3472-4)
Supplement: Supplementary file 1 — Interview protocol. (DOC 26 kb) [file 12913_2018_3472_MOESM1_ESM.doc]

# Interview Protocol

Project: **Improvement of the quality payment program by improving data reporting process**

Date ___________________________

Time ___________________________

Location ________________________

Interviewer ______________________

Interviewee ______________________

**Participants' Characteristics**

Gender: Female 🖵 Male 🖵

Age: ………………………..

Job: ……………………………

Years of Job experiences: ……………………

**Would you like to participate in this interview?**

Verbal Consent was obtained from the study participant

Verbal Consent was NOT obtained from the study participant

**Notes to interviewee**:

Thank you for your participation. I believe your input will be valuable to this research and in helping grow all of our professional practice. Obviously, the information contained in the questionnaire is completely confidential. Participation in the survey is completely voluntary and will not affect your score and course result. We sincerely thank and appreciate the fact that you are helping the researchers with honesty and openness in commenting. Approximate length of interview: 30 minutes, five major questions.

**Purpose of research:**

To establish the appropriate data reporting method in pay-for-performance program in Tabriz University of Medical Sciences.

**Questiona**

1. What is the appropriate method of data reporting in P4Q program and how does it would be conducted well?
2. What weaknesses and strengths did you experienced about the implemented (excel) method of data reporting in P4Q program?
3. What are your suggestions to overcome the weaknesses?
4. What weaknesses and strengths did you experienced about the implemented web-based method of data reporting in P4Q program?
5. What are your suggestions to overcome the weaknesses?

***And so forth***
