# Supplementary material for: Improvement of the quality payment program by improving data reporting process: an action research
Source: BMC Health Serv Res. 2018 Sep 6;18:692. doi: 10.1186/s12913-018-3472-4 (PMC6128004; doi:10.1186/s12913-018-3472-4)
Supplement: Supplementary file 2 — Informed consent letter. (DOC 42 kb) [file 12913_2018_3472_MOESM2_ESM.doc]

**Improvement of the quality payment program by improving data reporting process: an action research**

Dear Participant,

We are conducting this research project by the aim of establishment of the data reporting method in P4Q program which no long is initiated in primary health setting. I believe your input will be valuable to this research and in helping grow all of our professional practice. We sincerely thank and appreciate the fact that you are helping the researchers with honesty and openness in commenting. Following we provide you information about the introduction about the project, ethical considerations, purpose of the study, questions, and researchers brief characteristics.

**Introduction**

Recently P4Q program has been initiated to manage the incentives payment to primary health care providers who are not eligible to earn extra money in family physician program. As you may know, in P4Q program payment of incentives to healthcare providers are implemented based on a formula. The formula encompasses five main elements: 1- fixed payment, 2-performance score of individual health care providers, 2- performance score of the health centers as a team, 4- organizational performance, and 5-managerial appraisal. Each one f these elements have certain weight (for more information please see the instructions of P4Q program in East-Azerbaijan). However, at this research project we are looking for the appropriate methods to calculate the amounts of payment for each healthcare provider according to the formula.

**Ethical Consideration**

- We assure that your participation in this study is quietly voluntarily and you can leave the study any time and in each step of the study if you want.
- We assure that all of your data will be anonymous and nobody will access them by the identifiable information.
- We assure that we will confirm confidentiality in data collection, data analysis and reports.
- We assure that all data will be coded and will be used by code in the analysis and reports.
- We assure that your participation in this study will not have any harm for you or your relatives.
- We assure that avoid to participate in this study will not have any consequence on your work, relationships, or opportunities.
- We assure that this study is conducted in Research Deputy of Tabriz University of Medical Sciences.
- The reports of the study will send for you if you request.

**Purpose of research:**

To establish an appropriate data reporting method in pay-for-performance program in Tabriz University of Medical Sciences based on stakeholders' viewpoints?

**Questions**

1. What is the appropriate method of data reporting in P4Q program and how does it would be conducted well?
2. What weaknesses and strengths did you experienced about the implemented (Excel) method of data reporting in P4Q program?
3. What are your suggestions to overcome the weaknesses?
4. What weaknesses and strengths did you experienced about the implemented web-based method of data reporting in P4Q program?
5. What are your suggestions to overcome the weaknesses?

***And so forth***

**Research team**

**SI**: A researcher in Tabriz University of Medical Science with Ph.D. degree in Health Services Management.

*Address*: Department of health services management, school of health services management and medical informatics, Tabriz University of Medical Science, Tabriz Iran.

**JT**: A faculty of Tabriz University of Medical Science with MD and Ph.D. degree in Health Services Management. He has around 25 years experiences in Public health system.

*Address*: Department of Health Services Management, school of health services management and medical informatics, Tabriz University of Medical Science, Tabriz Iran.

**AG**: Research assistant of Health Services Administration in University of Alabama at Birmingham.

*Address*: Department of Health Services Administration, School of Health Professions, The University of Alabama at Birmingham, US.

**MF**: An expert in provincial primary health center of Tabriz University of Medical Science with MD degree.

*Address:* Health deputy of Tabriz University of Medical Science, department of research. Tabriz, Iran.

**KG**: A faculty of Tabriz University of Medical Science with Ph.D. degree in health services management.

*Address*: Iranian Center of Excellence in Health Management, Department of Health Service Management, Tabriz University of Medical Sciences, Tabriz, Iran.
